# A distinction between two instruments measuring dispositional mindfulness and the correlations between those measurements and the neuroanatomical structure

**DOI:** 10.1038/s41598-017-06599-w

**Published:** 2017-07-24

**Authors:** Kaixiang Zhuang, Minghua Bi, Yu Li, Yunman Xia, Xuehua Guo, Qunlin Chen, Xue Du, Kangcheng Wang, Dongtao Wei, Huazhan Yin, Jiang Qiu

**Affiliations:** 1 Key Laboratory of Cognition and Personality (SWU), Ministry of Education, Chongqing, China; 2 Department of Psychology, Southwest University, Chongqing, China; 3 Beibei Mental Health Center, Chongqing, China; 4 School of Education, Hunan Normal University, Hunan, China

## Abstract

The most widely used measurements of mindfulness are the Mindful Attention Awareness Scale (MAAS) and the Five Facet Mindfulness Questionnaire (FFMQ). However, controversies exist regarding the application of these scales. Additionally, the neural mechanisms of dispositional mindfulness havebecome a topic of interest. In the current study, we used surface-based methodology to identify the brain regions underlying individual differences in dispositional mindfulness in a large non-clinical sampleand compared the two instruments for measuring the dispositional mindfulness. The results indicated that theMAAS scores were significantly associated with increased greymatter volumes in the right precuneus and the significant association between the precuneus and depression symptomatology was mediated by MAAS scores. Regarding the FFMQ, the Describing, Nonjudging, and Nonreactivity facets were selectively associated with the cortical volume, thickness and surface area of multiple prefrontal regions as well as the inferior parietal lobule. Importantly, Describing mediated the association between the dorsolateral PFC volume and the cognitive reappraisal strategies of emotion regulation. These resultssuggested that the MAASwere mainly associated with self-awareness, while the FFMQ facets were selectively involved in emotion regulation, attention control and self-awareness. Therefore, this study characterized the differences in inter-individual variability between the two typical measurements of dispositional mindfulnessand the correlations between those measurements and imaging analyses.

## Introduction

The mediation of mindfulness has been increasingly incorporated into multiple psychotherapeutic interventions to relieve psychosomatic, psychiatric symptoms and to improve health-related quality of life^1, 2^, especially over the last two decades. Mindfulness originally stems from Buddhism^3^, a result of more than 2,500years of development, and can be described as a phenomenological approach oriented towards a gradual understanding of direct experience^4^. Within this Buddhist perspective, mindfulness meditation is typically characterized as an attribute of open-hearted awareness of moment-to-moment perceptible experience without judging^5^. This description encompasses attention and acceptance, which inherently refers to cognitive, attitudinal, affective, and even social and ethical dimensions. Furthermore, mindfulness can be considered dynamically rather than a single state. It is an active, investigative and attentive practice or process that differs between persons. Moreover, ordinarily, it can be enhanced through mindfulness-based systematic training with gradual refinement. However, although mindfulness-based training, such as integrative body-mind training (IBMT), mindfulness-based stress reduction (MBSR), has been burgeoning in recent years^6^, little is known regarding the individual differences of dispositional mindfulness. As Tang, Hölzel and Posner emphasized in correspondence in reply to acomment on their overview of the neuroscience of mindfulness^6, 7^, the neuro-behaviour changes induced by mindfulness practice maybe influenced due to pre-existing differences in dispositional mindfulness. Nevertheless, it is important to investigate dispositional mindfulness for assessing the effects of mindfulness meditation and for distinguishing between dispositional mindfulness and intentional mindfulness.

Dispositional mindfulness is generally measured by self-report inventories, and so far, the most widely used measurements are the Mindful Attention Awareness Scale (MAAS)^8^ and the Five Factor Mindfulness Questionnaire (FFMQ)^9^. The MAAS has been proven as a signal dimension^10^ scale that operationally defines mindfulness as “an openorreceptive attention to and awareness of ongoing events and experience^11^”. Brown and Ryan restrict mindfulness to present awareness control that subsumes an attitude of accept ancetowards one’s experience. The MAAS shows a good range of internal consistency a (Cronbach alphas ranging from 0.80 to 0.87) and high test re-test reliability (ICC = 0.81). Consistent with expectations, the construct validity of the MAAS was evidenced by negative correlations with self-report measures of depression, anxiety and rumination, and positive correlations with well-being, openness, pleasant affects and self-esteem^8, 12^. Nonetheless, some studies have also been reported on adaptive query, where the convergent validity among other putative mindfulness instruments was weak tomoderate^9, 12^. Moreover, MAAS scores showed no significant difference between novice meditators and non-meditators^13^, nor between 15-year meditators and a normative convenience sample with any mindfulness meditationexperience^14^. Some studies have also reported that no significant improvement in MAAS scores were observed after MBSR^15, 16^.

On the other hand, the FFMQ encompasses five components of mindfulness: Observing (observation of one’s internal experience and sensations); Nonjudging (non-judging of inner experience); Describing (a tendencyor ability to put sensations, perceptions, thoughts, feelings, emotions, or experiences intowords); Nonreactivity (nonreactivityto inner experiences) and Acting withawareness (the ability to focus undivided attention versus on automatic pilot). The construct validity of the FFMQ was supported by negative correlations with alexithymia, psychological symptoms, neuroticism and difficulties in emotion regulation, and positive correlations with openness, emotional intelligence, and self-compassion^9, 17^. In particular, higher scores of Nonjudging predicted lower degrees of depression, anxiety, and stress-related symptoms, and higher scores of Acting With Awareness predicted lower levels of depressive symptomatology^18^. However, other evidence regarding the validity of the FFMQ has raised concerns. Researchers noted that Observing might have a poor predictive validity^19, 20^ and different meanings in variable samples (meditators and non-meditators, high- and low-worry groups)^21, 22^. Zen Buddhists have indicated that Describing does not capture the essential element of mindfulness well, and it was difficult to interpret the meaning of acting with awareness and nonjudging items^23^. Given the mixed and debatable empirical behaviour findings of the MAAS and the FFMQ, we found that the two measurements were both widely used to assess dispositional mindfulness but that they might engage different components of mindfulness.

The available neuroscientific literature has mainly focused on the intentionalpractice of mindfulness, although few studies have explored the association between neuroanatomical structure/brain activity and individual differences in dispositional mindfulness. For instance, a few magnetic resonance imaging studies observed that higher dispositional mindfulness, as assessed by MAAS, was associated with greater prefrontalcortical activation and less amygdala activity during the affect labelling task^24^, greater parietal activation during the breath task^25^, and lower late positive potentials (LPP)of event-related brain potentials to emotional visual stimuli^26^. Two studies found that dispositional mindfulness, as measured by the MAAS, was associated with grey matter volume (GMV) in many regions, such as the amygdala, the caudate (related with reduced stress and negative affectivity)^27^, the hippocampus, the anterior cingulate cortex, the fronto-limbic network, the posterior cingulate cortex, and the temporal-parietal junction (related to executive attention, emotion regulation, and self-referential processing)^28^. Only a small sample study assessed the dispositional mindfulness using the FFMQ and showed that the Describing facet was positively correlated with the GM volume of the amygdala andthe insula (related to parts of the somatic marker circuit)^29^. Currently, little is known regarding the neuroanatomical substrates in the GM volume of dispositional mindfulness, and moreover, it remains unknown whether and how cortical thickness and surface area could predict dispositional mindfulness. As we expected, the results of this study regarding neuroanatomical structures are inconsistent for the MAAS and the FFMQ. Based on previous studies^27–29^, we hypothesized that the MAAS might be associated with brain structures involved in attention control, emotion regulation and self-referential processing and that subscales of the FFMQ might be associated with brain structures engaged in emotion regulation and self-awareness. Then, we used surface-based morphometry to investigate the relationships among cortical thickness, volume, surface area and individual differences in the MAAS and FFMQ within a large sample of healthy adults. We separately examined the specific mechanisms of individual differences in anatomical structure based on mediation analysis and whether mindfulness exerts beneficial effects on depressive vulnerability and emotion regulation. We expected this study to support the use of the MAAS and FFMQ in various mindfulness-based training and therapeutic studies, such as assisting investigators in choosing the optimal questionnaire for their study, to emphasize the corresponding components of mindfulness. We envision that dispositional mindfulness plays a productive role in depression vulnerability and emotion regulation strategies.

## Results

### Sample descriptive statistics

**Figure 1:**
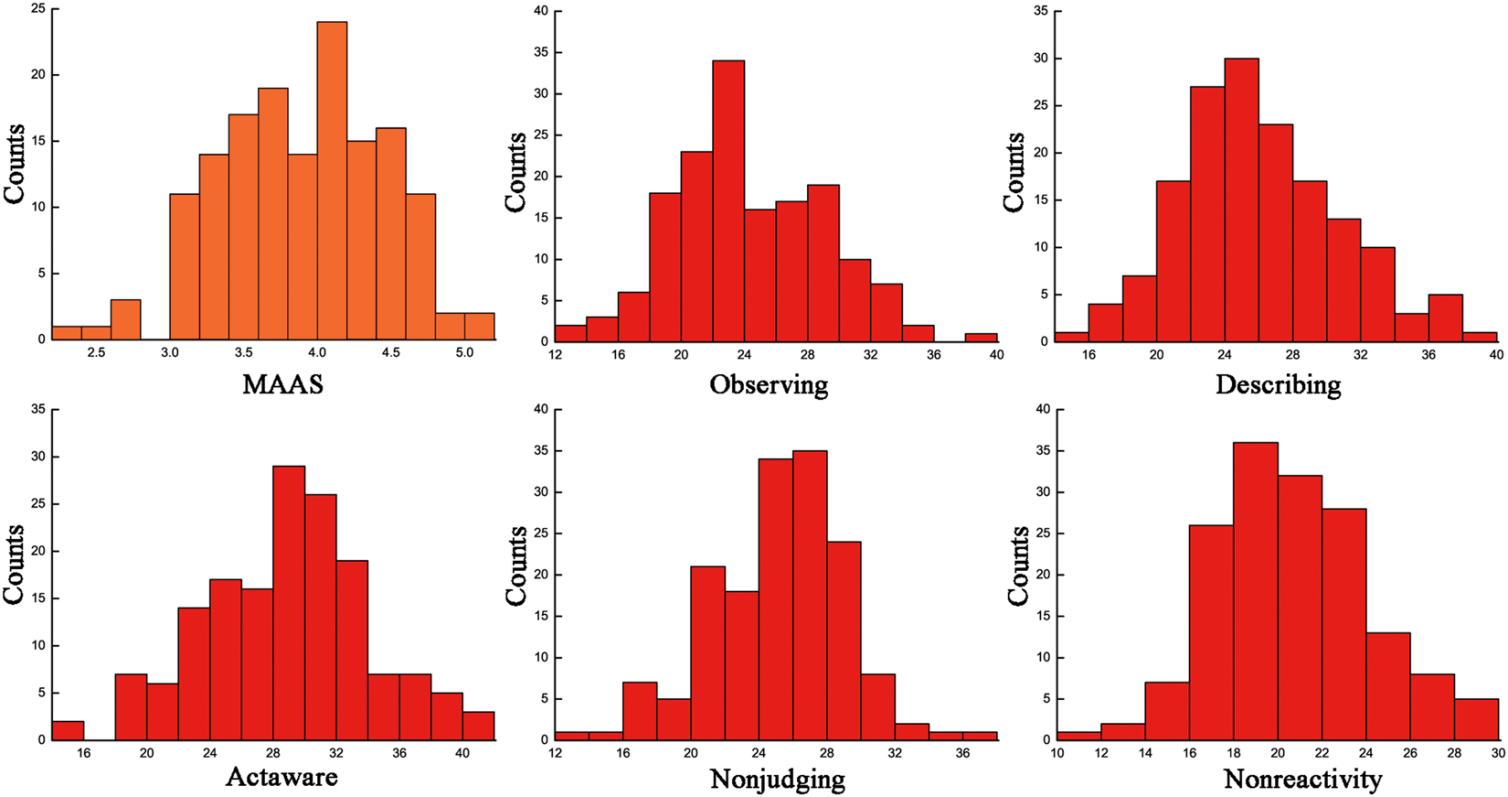
Distribution of the scores from MAAS and the five dimensions of FFMQ in our sample.

Table 1 shows the characteristics of the demographic variables of all subjects and the Pearson correlation coefficients for the MAAS and the five dimensions of the FFMQ. As indicated in Table 1, the MAAS score was negatively correlated with depression (r = −0.28, P < 0.01), and the Describing facet was positively associated with cognitive reappraisal (r = −0.30, P < 0.05). These correlations represent the fundamentals of the meditation analysis along with seven other correlations (see Table 1 for details). For complete data on the distribution of MAAS and FFMQ, see Fig. 1. These results promote interpretation of the following correlations.

**Table 1:** Demographics and psychometric measures.

	MAAS	Observing	Describing	Actaware	Nonjudging	Nonreactivity
Means	3.89	23.86	25.56	28.35	24.50	20.23
SD	0.55	4.73	4.65	5.20	3.95	3.46
Age	19.68 (2.07)^a^	21.72 (1.03)	/	/	/	/
Sex	F = 90, M = 60	F = 101, M = 57	/	/	/	/
BDI	−0.28**	−0.02	−0.25*	−0.40**	−0.29**	−0.05
ERQ-CR	0.15	0.19*	0.30*	0.12	−0.18*	0.06
ERQ-ES	−0.08	0.15	−0.05	−0.10	−0.19*	0.18*

SD = standard deviation; BDI = Beck Depression Inventory; ERQ-CR = Cognitive Reappraisal dimension of Emotion Regulation Questionnaire; ERQ-ES = Expressive Suppression dimension of Emotion Regulation Questionnaire. Pearson bivariate correlations, where the r-values are shown; ^a^mean ± SD; *P < 0.05; **P < 0.001.

### Surface-based morphometry results

**Figure 2:**
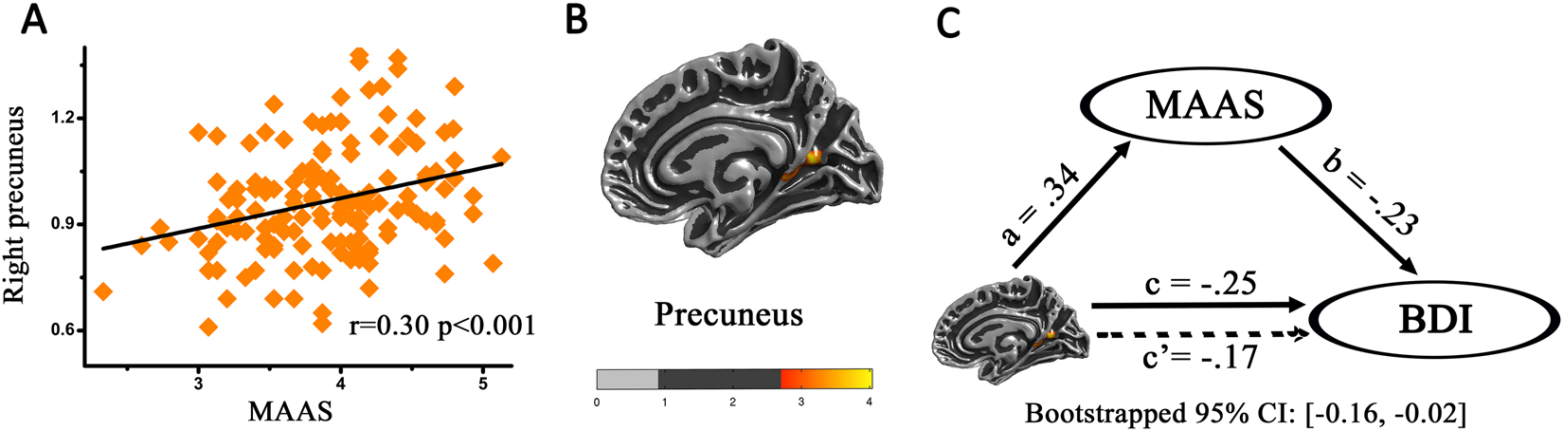
Cortical volume correlated with MAAS scores. (**A**) The scatter plot shows the pattern of correlation between MAAS scores and the right precuneus. (**B**) The right precuneus exhibited a significant positive correlation with MAAS scores. (**C**) Mediation analysis: Path c is the total effect of the depression symptoms on the precuneus; path c’ is the direct effect of the depression symptoms on the precuneus; after controlling for MAAS scores, the product of the paths a and b (ab) is the indirect effect of the depression symptoms through MAAS on the precuneus.

**Figure 3:**
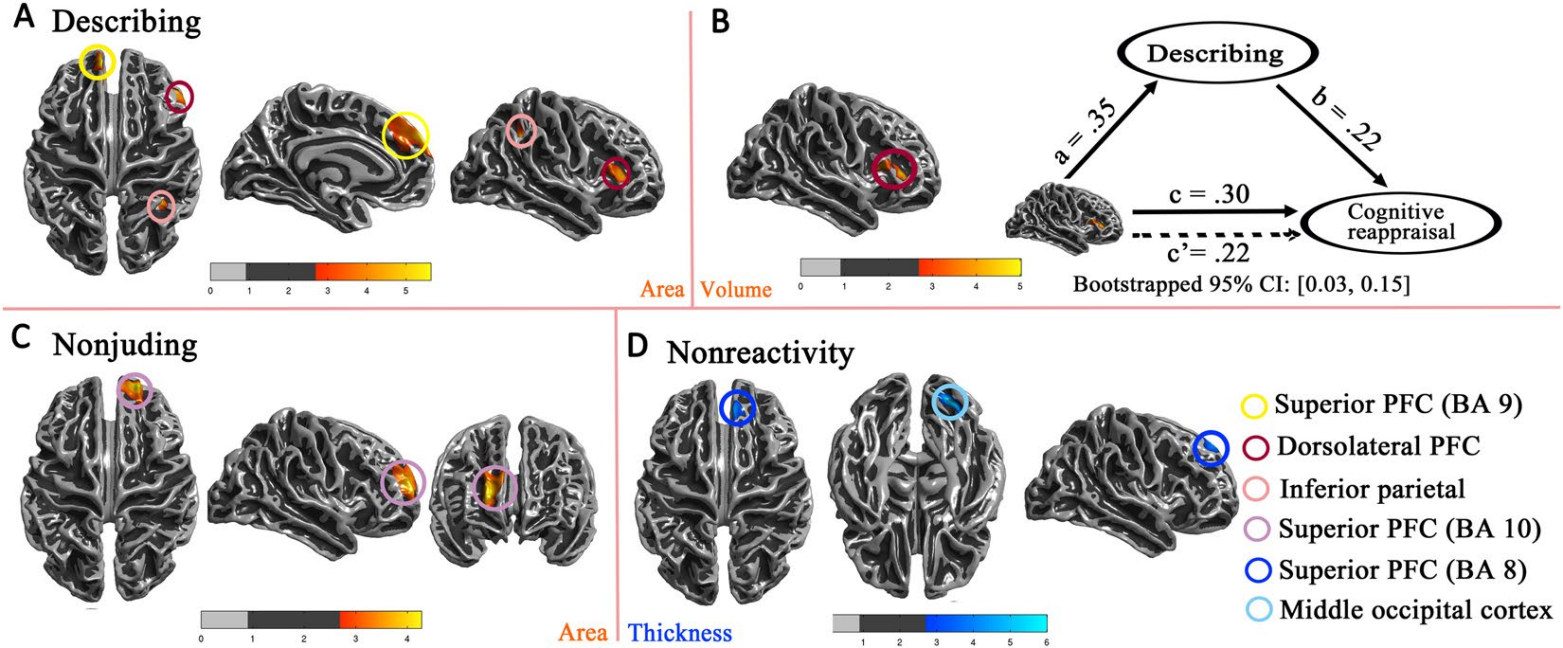
Cortical thickness, volume and surface area were correlated with the FFMQ. (**A**) The surface areas of the superior PFC (BA 9), the dorsolateral PFC (BA46) and the inferior parietal lobule exhibited significant positive correlations with the Describing facet. (**B**) The cortical volume of the dorsolateral PFC (BA 46) was positively correlated with the Describing facet, and the Describing mediated the association between the dorsolateral PFC and cognitive reappraisal. (**C**) The surface area of the superior PFC (BA 10) was significantly correlated with the Nonjuding facet. (**D**) Superior PFC (BA 8) thickness exhibited significant negative correlation with the Non reactivity facet.

In this study, we investigated the cortical volume, thickness and surface area associated with individualdifferences in dispositional mindfulness corresponding to the MAAS and FFMQ scores. Multiple regression analysis revealed that MAAS scores were significantly and positively associated with the corticalvolume in the right precuneus (Fig. 2, see Table 2 for correspondingbrain coordinates). Regarding the FFMQ, the Describing facet was significantly correlated with the surface area in the right dorsolateral prefrontal cortex (PFC) (Brodmann area, BA 46), the inferior parietal lobule (BA 40) and the left superior PFC (BA 9) (Fig. 3A, see Table 2 for correspondingbrain coordinates). Moreover, the dorsolateral PFC was also significantly observed in the Describing facet with surface area (Fig. 3B). The Nonjudging facet was positively correlated with surface area in the superior PFC (BA 10) (Fig 3C). Interestingly, the Nonreactivity facet was associated with reduced cortical thickness in the right superior PFC (BA 8) and the middle occipital cortex (Fig. 3D). All the results were corrected for RFT at the whole-brain level of P < 0.05 (voxel level P < 0.001).

**Table 2:** Brain coordinates corresponding to cortical volume, volume and surface area in Figs 2 and 3.

Indexes	Facets	Brain region	Right/Left	Peak (Talairach)			T
				X	Y	Z	
Volume	MAAS	Precuneus	R	22	−57	12	4.04
	Describing	Dorsolateral PFC (BA 46)	R	44	34	3	5.02
Area	Describing	Dorsolateral PFC (BA 46)	R	46	31	6	4.66
		Inferior parietal lobule (BA 40)	R	35	−45	36	4.89
		Superior PFC (BA 9)	L	−8	56	26	5.64
	Nonjudging	Superior PFC (BA 10)	R	21	57	12	4.27
Thickness	Nonreactivity	Superior PFC (BA 8)	R	8	44	40	−4.28

### Mediation analysis

Given the importance of mindfulness in depression and emotion regulation, which has been studied in a wide range of mindfulness-based training studies, we performed a mediation analysis to test whether dispositional mindfulness plays a protective role in alleviating depression symptoms and regulating emotions based on a neural mechanism. Evidence from the partial correlation analyses (controlling for age and gender) indicated that there were nine significant correlations representing the fundamentals of the meditation analysis. However, after the mediation evaluation, we found only two significant results. First, the MAAS scores mediated the association between the precuneus and depression symptoms (Fig. 2C). Moreover, the Describing scores also mediated the relationship between the dorsolateral PFC and cognitive strategies (Fig. 3B). All mediation analyses were based on a script (http://afhayes.com/spss-sas-and-mplus-macros-and-code.html#modmed) written by Andrew F. Hayes of the Ohio State University (Preacher&Hayes, 2008).

## Discussion

In this study, we explored the correlations between dispositional mindfulness and brain structures in a large sample of non-clinical young adults and compared the two instruments measuring the dispositional mindfulness. We found that MAAS scores were significantly associated with greater GM volume in the right precuneus. Regarding FFMQ scores, the Describing facet was significantly associated with greater surface area in the right dorsolateral PFC (BA 46), the inferior parietal lobule (BA 40) and the left superior PFC (BA 9) and GM volume in the dorsolateral PFC (BA 46). The Nonjudging facet was associated with greater surface area in the superior PFC (BA 10). Additionally, the Nonreactivity facet was associated with reduced cortical thickness in the right superior PFC (BA 8) and the middle occipital cortex. Furthermore, we performed a mediation analysis, which showed that dispositional mindfulness (MAAS scores) mediated the relationship between structural variations in the precuneus and depression vulnerability. Similarly, dispositional mindfulness (Describing facet) mediated the association of the dorsolateral PFC and cognitive reappraisal. The mediation analysis further confirmed that dispositional mindfulness might play a crucial role in the interaction between depression symptoms and brain regions. These results suggest that dispositional mindfulness as measured by MAAS scores is mainly engaged in self-awareness, while dispositional mindfulness as measured by the FFMQ is involved in attention control, self-awareness and emotion regulation, and the mediation analysis of the precuneus/dorsolateral PFC helps explain the broad beneficial effects exerted by mindfulness on depression symptoms and the cognitive reappraisal strategies of emotion regulation.

The correlation between MAAS and GM volume in the precuneus supports the basic definition of MAAS –“an open or receptive attention to and awareness of ongoing events and experience”^11^–which emphasizes self-awareness^6^ as one of the important components of mindfulness meditation. It has been suggested that the precuneus is involved in the brain network of the neural correlates of self-awareness^30, 31^, or more specifically, it concerns self-referential mental representations^32, 33^ and awareness of present-moment experiences. The precuneusas a distinct hub characterizing the default mode network seems to be engaged in the internal processes of mental images and spontaneous thoughts^34^ and in non dual awareness^35^. A previous study revealed that the interactivity of the precuneus and the prefrontal cortex has been regarded as involving the state of reflective self-awareness^36^. Moreover, the interaction between the precuneus and fronto polar regions in visual stimuli indicates that the precuneus is engaged in state-dependent awareness^37^. In addition, the mediation effect of mindfulness on the relationship between the precuneus and depression symptoms might indicate that mindfulness influenced the correlation between depression symptoms and the precuneus area. Converging our results and evidence from previous studies, MAAS-corresponding mindfulness meditation might influence the precuneus, which is engaged in self-awareness, especially in self-referential processing^38, 39^ and moment-to-moment experiences.

Regarding the FFMQ, multiple prefrontal regions (Describing&Nonjuding: superior PFC (BA 9 and 10); Nonjudging: superior PFC (BA 8); Describing: dorsolateral PFC), the inferior parietal lobule and the middle occipital gyrus were significant. These regions play key roles not merely in self-awareness but also in attention control and emotion regulation, which are two other primary components of mindfulness meditation^40, 41^. A detailed discussion is presented below.

First, increases in both GM volume and surface area of the dorsolateral PFC (BA 46) were observed corresponding to the Describing facet. As a classical hub location in the central executive network, the dorsolateral PFC (after 6 weeks of mindfulness training) was observed to exhibit increased activity during executive processing in response to an affective Stroop task involving attentional control and affective processing^42^. Likewise, a recent study indicated that, after 8 weeks of mindfulness training, the right dorsolateral PFC displayed greater activation, which is an area engaged in sustaining and monitoring the focus of attention^43^. The dorsolateral PFC also exhibited increased activation while viewing passive images within a mindful state^40, 44^. Moreover, the Describing facet mediated the association between the dorsolateral PFC and cognitive reappraisal strategies. Given these results, the describing facet of dispositional mindfulness might exhibit an enhanced level of top-down attention and improved emotion regulation through the dorsolateral PFC, which is linked to a conflict resolving mechanism.

Second, we detected a positive correlation between the Describing facet and the surface area of the left superior PFC (BA 9) and between the Nonjudging facet and the surface area of the right superior PFC (BA 10). In an exploratory analysis^45^, greater GM densities were observed in BA 10, which is crucial for self-referential processing^46^ throughintrospection activity and thereby self-awareness. In a comparison study^47^ of brain structures between long-term insight meditators and matched controls, the meditators exhibited significantly thicker and slow age-related thinning cortical thickness in BA 9 and 10, which have been proposed to integrate emotion and cognition^48, 49^. It has also been demonstrated that BA 9 and 10 showed increased activation while viewing passive images within a mindful state^40, 42^. Given the evidence reported in these studies, the superior frontal cortex (BA 9 and 10)might play crucial roles in emotion regulation, sensory information and internal bodily sensations. Interestingly, we found that the superior frontal cortex (BA 8)/middle occipital cortex were negatively correlated with Non reactivity in the individual difference analysis of cortical thickness. A surface-based morphometry study reported that meditators, compared to controls, displayed thicker cortical thickness in the frontal-temporal region, including the superior frontal cortex, and thinner thickness in the posterior portions of the brain, including the middle occipital cortex^50^. Studies have also suggested that GM volume undergoes nonlinear changes, with increases in training length andintensity^51^, which increased during the initial stage and reduced in more advanced stages^52, 53^. Consequently, we speculate that the high level of Non reactivity to inner experiences might be associated with high degrees of openness and acceptance of negative views while corresponding to low regulation effort and mental control, although further studies are needed to examine this hypothesis. The present findings of the superior PFC indicate that each facet, which correspond special prefrontal regions that selectively engage in self-awareness (especially regarding sensory information and internal bodily sensations) and emotion regulation, may constitute an important part of dispositional mindfulness.

A significant association between the inferior parietal lobule (BA 40) and the describing facet of mindfulness was identified in surface area. The inferior parietal lobule (BA 40), a part of Wernicke’s area, was correlated with language ability development^54^, which is in accordance with the basic definition of the Describing facet and the tendency of describing the verbalization of emotions into words. As a major node of the mindfulness meditation complex network, especially in relation to fronto-parietal networks^55^, the inferior parietal lobule performed significantly increased activity during meditation^56^ or after training^38^. For example, 4-year experienced meditators showed greater inferior parietal cortex activity during a brief meditation compared with a control state^56^, and there were also meditators compared to controls who showed increased blood flow^57^ and activation^58^ of the inferior parietal lobule, which is regarded to be involved in attention control. After mindfulness-based stress-reduction, social anxiety disorder patients^59^ displayed a greater BOLD response in the inferior parietal lobule, which might specifically be related to alerting^41^ componentsof visual-related attention. Similarly, trained health meditators displayed increased recruitment of inferior parietal cortices^38^ during a momentary self-reference task (experiential versus narrative). Furthermore^60^, the inferior parietal lobule is considered as central to the subjective feelings that might consume the effort of the executive control of attention to be mindful. This also indicates that the inferior parietal lobule plays a special role in present-focused awareness with transitory experiences. Moreover, an emotion strategy comparison study revealed that the inferior parietal lobule was activated when subjects used a mindfulness strategy to view negative pictures compared with the no strategy condition^61^. Considering these investigations, the interaction between Describing and the inferior parietal lobule might be engaged inattention control (specifically related to alerting components), self-awareness and negative affection regulation.

One limitation of the current study is that the relationship between mindfulness constructs and structural neuroanatomical properties might not apply to psychiatric populations. As only healthy subjects were included in this study, the generalization of our findings is restricted. Therefore, it is uncertain whether psychiatric patients exhibit similar dispositional mindfulness. This issue remains to be elucidated in future investigations. In summary, the current study assessed cortical volume, thickness and surface area to contrastively explore the individual differences in surface structures in two typically and widely used tests of mindfulness within a large sample of healthy adults. MAAS scores were positively correlated with the cortical volume of the GM in the precuneus. On the other hand, the FFMQ recruited multiple prefrontal regions, the inferior parietal lobule and the middle occipital cortex, for which cortical volume, thickness and surface area corresponded to various facets. The results of MAAS and the FFMQ indicate that the MAAS scores were mainly associated with self-awareness, which is concentrated on self-related experiences and maintainingone’s awareness of experiences moment-to-moment, while various facets of the FFMQ are selectively involved in self-awareness, attention control and emotion regulation. This study contributes to efforts to characterize differences in inter-individual variability between the two typical measurements of dispositional mindfulness, provides exploratory results of the brain for reference, and offers evidence for aiding in determining which measurement is optimal for assessing mindfulness in further studies. Finally, further studies should investigate the individual differences in dispositional mindfulness after various mindfulness-based trainings and investigate the changes after intervention. In particular, longitudinal studies should be conducted. It is important to investigate the precise mechanism of how pre-dispositional mindfulness affects deliberate mindfulness and then to distinguish healthy subjects or patients who are most sensitive to mindfulness interventions for clinical applications.

## Methods

### Subjects

All subjects were from Southwest University, China, and participated in this study as part of anongoing project to examine theassociations among brain imaging, creativity and mental health. In total, 154 subjects finished the MAAS assessment, but 4 subjects were excluded who fell outside of three standard deviations of the mean. In addition, 162 subjects finished the FFMQ, but 3 subjects were excluded who had scores that were more than three standard deviations, and 1 subject was excluded due to damage to the brain images. All subjects were right-handed and had no neurological or psychiatric disorders or substance use (including illicit drugs and alcohol). According to the Declaration ofHelsinki^62^, written informed consent was obtained from all participants prior to engaging in the research tasks. All study procedures were performed in accordance with relevant guidelines approved by the responsible committee for experiments involving human subjects of the Southwest University Brain Imaging Center Institutional Review Board. Importantly, before executing the experiment, the experimental protocols of behaviour and brain were approved by the Southwest University Brain Imaging Center Committee.

### Assessment of dispositional mindfulness

Mindfulness was measured by the Mindful Attention Awareness Scale (MAAS)^8^ and the Five Factor Mindfulness Questionnaire (FFMQ)^9^. The MAAS focuses on attentiveness and awareness of the present-moment, such as “I tend not to notice feelings of physical tension or discomfort until they really grab my attention” and “I find myself preoccupied with the future or the past”. The MAAS is an unidimensional, self-report questionnaire including 15 items and is associated with superior internal consistency (α = 0.82) and test-retest reliability (r = 0.81)^8^. Additionally, the MAAS uses a six-point rating scale ranging from 1 (almost always) to 6 (almost never), where higher scores reflect a higher tendency to be mindful in daily life.

The FFMQ is a 39-item self-report questionnaire containing 5 dimensions of mindfulness: Observing, Describing, Acting with Awareness, Non-Reactivity and Nonjudging^9, 19^. There is evidence of the internal consistency of the five subscales (Cronbach alphas ranging from 0.67 to 0.93). The questions use a five-point Likert-type scale ranging from 1 (never or very rarely true) to 5 (very often or always true). The alpha coefficients of each subscale range from 0.75 to 0.91^9^, which provide sufficient evidence for internal consistency. In general, the scores of each subscale are used for interpretation, and higher scores indicate higher levels of dispositional mindfulness.

### Assessment of depression

The degree of depressive symptomatology was measured by the Beck Depression Inventory (BDI)^63^. The BDI is a 21-question self-report inventory that was developed to detect, evaluate, and monitor changes in depressive symptoms among individuals in mental health care settings^63^. The BDI is a 4-pointLikert-type scale (0–3), scores can range from 0 to 63, and higher scores reflect higher degrees of depression-like symptoms. This self-report measure is considered to have good reliability and validity in both clinical and nonclinical populations, including the Chinese version.

### Assessment of emotion regulation

Emotion regulation reflects the ability of taking action to either maintain or alter the intensity of emotion or to either prolong or shorten an emotional experience. Individual differences in emotion regulation ability was assessed by the Emotion Regulation Questionnaire (ERQ)^64^. The ERQ focus on two specific strategies of down-regulating emotion control: cognitive reappraisal and expressive suppression. The expressive suppression dimension contains four items, such as “I keep my emotions to myself” and “When I am feeling positive emotions, I am careful not to express them”. The cognitive reappraisal dimension is thought of as an antecedent-focused strategy, containing items such as “When I want to feel less negative emotion, I change the way I’m thinking about the situation”. For each item, subjects were instructed to indicate the extent to which they agree or disagree. Each item was rated on a 7-point Likert-type scale ranging from strongly disagree (1) to strongly agree (7). A high score of ERQ indicates frequent use of reappraisal/suppression strategy to decrease individual’s emotional impact. Moreover, The ERQ has high internal consistency, test-retest reliability, and convergent validity^64^.

### Structural MRI acquisition

Scanning was performed using a Siemens 3 T scanner (MAGENTOM Trio, a Tim system) with a 12-channel phased-array head coil at the Southwest University Brain Imaging Center, Beibei, China. High-resolution T1-weighted structural images were acquired using a magnetization-prepared rapid gradient echo sequence: repetition time = 1900 ms; echo time = 2.52 ms; inversion time = 900 ms; flip angle = 9 degrees; resolution matrix = 256 × 256; slices = 176; thickness = 1.0 mm; voxel size = 1 × 1 × 1 mm.

### Surface-based morphometry

The details of structural MRI processing are described in the following subsections: removal of non-brain tissue by an improved hybrid watershed/surface deformation^65^; automated Talairach transformation; tissue segmentation of the subcortical white matter, the cerebrospinal fluid and deep GM; image intensity in homogeneity correction^66^; a triangular mesh tessellation over the grey matter-white matter boundary; automated topological defect correction on the surface; a surface deformation following intensity gradients to produce the grey-white matter interface and grey matter-cerebrospinal fluid interface; individual surface inflation; individual surface (volume) normalization to a spherical atlas^67^; divisionof the cerebral cortex intovarious regions respecting gyral and sulcal structuralinformation^68^; and creation of various surface-based datasets, such as maps of curvature and sulcal depth. A Gaussian distribution with a full-width–half-maximum (FWHM) of 10 mm was used for surface smoothing.

### Statistical analysis

In the whole-brain analyses, the Freesurfer’ mri_glmfit was performed to fit a general linear model to explore the relationship between the dimensions of the FFMQ/MAAS and the cortical volume, thickness, and area. Sex and age were regarded as regressors of no interest when we identified regions in which cortical structures were associated with individual differencesin MAAS scores. Importantly, when we performed an exploratory analysis of the relationships between the cortical structures and individual differences in each FFMQ dimension, we included age, sex and the other dimensions of FFMQ as covariates. This procedure generated statistical parametric maps that could be thresholded. Moreover, the statistical maps were thresholded using random field theory (RFT), and significant clusters of P < 0.05 (voxel level P < 0.001) were reported.
